# Single cell electroporation for longitudinal imaging of synaptic structure and function in the adult mouse neocortex *in vivo*

**DOI:** 10.3389/fnana.2015.00036

**Published:** 2015-04-07

**Authors:** Stéphane Pagès, Michele Cane, Jérôme Randall, Luca Capello, Anthony Holtmaat

**Affiliations:** ^1^Department of Basic Neurosciences and The Center for Neuroscience, Centre Médical Universitaire (CMU), University of GenevaGeneva, Switzerland; ^2^Itopie Informatique, Société CoopérativeGeneva, Switzerland

**Keywords:** Single cell electroporation, *in vivo*, long-term imaging, calcium imaging, dendritic spine

## Abstract

Longitudinal imaging studies of neuronal structures *in vivo* have revealed rich dynamics in dendritic spines and axonal boutons. Spines and boutons are considered to be proxies for synapses. This implies that synapses display similar dynamics. However, spines and boutons do not always bear synapses, some may contain more than one, and dendritic shaft synapses have no clear structural proxies. In addition, synaptic strength is not always accurately revealed by just the size of these structures. Structural and functional dynamics of synapses could be studied more reliably using fluorescent synaptic proteins as markers for size and function. These proteins are often large and possibly interfere with circuit development, which renders them less suitable for conventional transfection or transgenesis methods such as viral vectors, *in utero* electroporation, and germline transgenesis. Single cell electroporation (SCE) has been shown to be a potential alternative for transfection of recombinant fluorescent proteins in adult cortical neurons. Here we provide proof of principle for the use of SCE to express and subsequently image fluorescently tagged synaptic proteins over days to weeks *in vivo*.

## Introduction

Advancements in imaging techniques and recombinant fluorescent protein design have allowed the study of neuronal structures in the mouse neocortex *in vivo* (Denk and Svoboda, 1997; Miyawaki, 2005; Holtmaat and Svoboda, 2009). This has revealed that substrates of synapses, such as dendritic spines and axonal boutons are dynamic, i.e., they grow and shrink or appear and disappear, even in the adult cortex (Trachtenberg et al., 2002; De Paola et al., 2006; Holtmaat et al., 2006; Loewenstein et al., 2011). Although spines and boutons are considered to be reliable proxies for synapses, their presence does not correlate with synapses in a 1:1 fashion. Some spines, especially when they are less than one-day old, rarely contain a synapse (Knott et al., 2006; Arellano et al., 2007; Nägerl et al., 2007; Cane et al., 2014) and some boutons bear a synaptic contact with more than one spine (Sorra and Harris, 1993; Knott et al., 2006; Toni et al., 2007). Although alterations in synaptic strength have been shown to correlate well with short and long-term structural changes in organotypic slice cultures (Matsuzaki et al., 2004; Nägerl et al., 2004; De Roo et al., 2008a; Hill and Zito, 2013; Wiegert and Oertner, 2013), it is not clear how well-spine and bouton cytosolic volume dynamics report synaptic plasticity *in vivo*.

The most reliable measurement of synapse dynamics is obtained through direct imaging of molecular components of the pre- or postsynaptic complex (Okabe et al., 1999, 2001; Friedman et al., 2000; Becker et al., 2008; De Roo et al., 2008b; Woods et al., 2011). Fluorescently tagged postsynaptic scaffold proteins have been shown to accurately label synapses *in vivo*, which enables tracking of synapse dynamics (Gray et al., 2006; Chen et al., 2012; van Versendaal et al., 2012; Cane et al., 2014). Expression of synaptic proteins can be achieved through the electroporation of recombinant DNA vectors in embryonic primordial cortical neuroblasts (Saito and Nakatsuji, 2001; Tabata and Nakajima, 2001). Expression in these cells remains high upon differentiation, and can be visualized through a cranial window in the adult animal *in vivo* (Gray et al., 2006; Ako et al., 2011; Chen et al., 2012; van Versendaal et al., 2012). In most cases, expression is robust, starts immediately after birth, and occurs in a relatively large population of cells, which makes this technique useful for a large array of applications (Supplementary Table 1). However, the robust and widespread expression patterns often increase background fluorescence, which complicates *in vivo* imaging. Using conditional promoters and co-transfection, expression can be restricted to a sparse set of neurons (Ako et al., 2011; Chen et al., 2012). In addition, the perinatal expression of synaptic proteins, which possibly affects synaptic circuit formation and maturation can be avoided using such approaches (Ako et al., 2011). Finally, this technique does not allow to precisely target expression to a particular microcircuit, such as a single cortical column.

Recombinant viral vectors provide other advantages. However, it is difficult to tame expression levels and to precisely time the onset of expression. For certain viral vectors it may even take several weeks for expression to reach maximum levels (Supplementary Table 1). In addition, many viral vectors that are well-suited for transfection of adult cortical neurons (e.g., AAV) have limited packaging capacities. This complicates their use for expressing proteins that are encoded by long reading frames, such as some synaptic proteins (but see Mower et al., 2011 for a viral vector approach to express a synaptic protein).

Single cell electroporation (SCE) may offer an alternative method for the longitudinal study of cells *in vivo* (Haas et al., 2001; Rathenberg et al., 2003; Kitamura et al., 2008; Judkewitz et al., 2009). For this method, DNA vectors are electroporated in a single (or several) neuron(s) in the cortex *in vivo* using a glass pipette that is loosely attached to the neuron's membrane (Kitamura et al., 2008; Judkewitz et al., 2009). Upon electroporation, expression can usually be observed within 24 h, depending on the promoter driving the transcription (Supplementary Table 1). The electroporation can be applied to any cell type in the adult cortex and there is no strict limit to the size of electroporated plasmids. This technique has been used to transfect GFP (Kitamura et al., 2008; Judkewitz et al., 2009) or for trans-synaptic labeling (Rancz et al., 2011) in the mouse neocortex. When combined with the implantation of a chronic cranial window, this technique potentially provides a suitable preparation to study with high spatial and temporal resolution the dynamics of synaptic proteins in single adult cortical neurons over long times without disrupting synaptic circuits. Here, we have adopted the SCE method (Kitamura et al., 2008; Judkewitz et al., 2009) and combined it with the implantation of a chronic cranial window (Holtmaat et al., 2009) to express and image synaptic proteins over days to weeks *in vivo*. We provide a description of the methods, some examples of time-lapse imaging of synaptic proteins and function, and an analysis method for synaptic protein dynamics.

## Materials and methods

### Vectors

pCAG-DsRedExpress-WPRE and pCAG-PSD-95-eGFP-WPRE were obtained from Svoboda, Janelia Farm Research Campus (Gray et al., 2006; Cane et al., 2014). pCAG-eGFP-gephyrin-WPRE was obtained from Levelt and Schwarz (van Versendaal et al., 2012). pCAG-eGFP-CaMKIIα-WPRE was cloned from a plasmid obtained from Hayashi (Takao et al., 2005). pCAG-SEP-GluR1-WPRE was cloned from pCI-SEP-GluR1, obtained from Malinow (Kopec et al., 2006). hSyn1-mRuby2-GSG-P2A-GCaMP6s-WPRE plasmid was obtained from Rose and Bonhoeffer (Addgene plasmid # 50942).

### Intrinsic optical imaging, single cell electroporation, and cranial window implantation

These experiments were performed according to the guidelines of the Swiss Federal Act on Animal Protection and Swiss Animal Protection Ordinance. All experiments were approved by the ethics committee of the University of Geneva and the Cantonal Veterinary Office (Geneva, Switzerland). The SCE as described here has been adapted from Judkewitz et al. (2009) without major modifications. However, we sought to combine this with intrinsic optical imaging as well as the implantation of a cranial window that allows targeted and longitudinal imaging of neuronal structure or function. Therefore, at particular points we emphasized that what we thought is important for the successful combination of the three techniques.

Anesthesia was induced and maintained by an intraperitoneal injection of a mixture (MMF) containing medetomidin (Dorbene, 0.2 mg kg^−1^), midazolam (Dormicum, 5 mg kg^−1^) and fentanyl (Sintenyl, 0.05 mg kg^−1^) in sterile NaCl (0.9%). To prevent potential inflammation, bradycardia, or salivary excretions, carprofenum (Rimadyl, 5 mg kg^−1^) and glycopyrrolate (Robinul, 0.01 mg kg^−1^) were injected subcutaneously before surgery.

**Figure 1:**
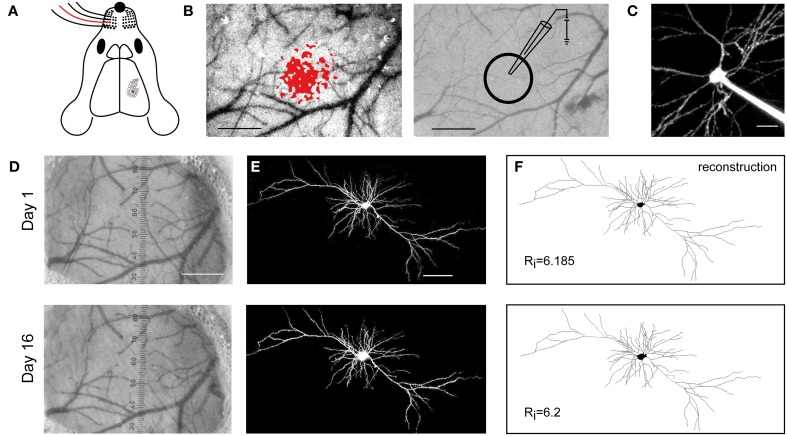
**Long-term expression and viability upon SCE**. **(A)** Schematic showing the mapping of the C2 whisker (red) onto the barrel cortex. **(B)** Left, localization of the cortical representation of the C2 whisker, using intrinsic optical signal imaging. Right, the pipette for electroporation is targeted to L2/3 of the mapped cortical area. Scale bar, 500 μm. **(C)** A targeted cell, filled with Alexa 488 upon electroporation. Scale bar, 20 μm. **(D)** The blood vessel pattern remains stable between day 1 and 16 following the electroporation. Scale bar, 500 μm. **(E)** Two photon images of SCE-mediated co-expression of DsRedExpress on day 1 and 16. Scale bar, 50 μm. **(F)** Reconstructions of the cell in **(E)**, showing that the large-scale morphology of the cell remains unaltered. Rj, Schoenen Ramification Index.

The mouse head was fixed using a head holder (Narashige). The skin was disinfected with betadine and lidocaine (1% v/v) was injected under the skin, which was subsequently gently removed from the top of the skull. Sterile artificial cerebrospinal fluid (ACSF; in mM: 125 NaCl, 5 KCl, 10 D-Glucose, 10 HEPES, 1 Ascorbic Acid, 2 CaCl_2_, 2 MgSO_4_) was applied to a well that was constructed out of dental cement just above the somatosensory cortex (coordinates from bregma: Rostro-caudal: −1.5 mm, Latero-medial: 3.5 mm). This was covered with glass in order to keep the skull moist and transparent. Next, the mouse was transferred to the intrinsic optical signal setup and intrinsic signals were recorded as described previously (Figures 1A,B) (Gambino and Holtmaat, 2012). In short, responses as evoked by C2 whisker deflections were imaged using an Imager 3001F (Optical Imaging, Mountainside, NJ) equipped with a CCD camera and a halogen light source filtered at 700 nm. An image of the brain's surface vasculature was taken using green light (546-nm bandpass filter). The image of the intrinsic signal was superimposed over the vasculature image. This was used as a reference for the position of the craniotomy and the electrode for SCE.

The mouse was transferred back to the surgery setup and head fixed using a head holder. A small craniotomy was performed using an air driven dental drill just above the area that showed the maximum IOS response. The craniotomy was performed as described earlier (Holtmaat et al., 2009). The dura was left intact and care was taken not to cause any bruising. The craniotomy was adjusted to the size of the prospective cranial window (3 mm diameter). After the surgery the craniotomy was covered with sterile gel foam soaked in cortex buffer to keep the dura moist.

The mouse was transferred to a 2-photon laser-scanning microscope (2PLSM). Glass pipettes (15–20 MΩ) were pulled from glass capillaries (Harvard Apparatus GC150F-7.5) on a vertical pipette puller (Narishige PC-100). The backs of the pipettes were fire polished. Pipettes were filled with 1–2 μl of internal solution (composed of, in mM: 266 KMeSO_4_, 14 KCl, 20 Na-HEPES, 4 MgATP, 4 Na_2_ATP, 1 Na_2_GFP, and 0.1 EGTA; pH 7.2; 280–290 mOsm), containing a mixture of plasmids (final concentration: 30–200 ng μl^−1^) and Alexa Fluor 488 hydrazide (50 μM; Life Technologies). Before filling, the solution was filtered using a 0.45-μm centrifugal filter (Ultrafree-MC-HV, Millipore).

For electroporation we used an Axoporator (800A) and a headstage (AP-1AX1MU, Molecular Devices), attached to a micromanipulator (LN Junior, Luigs-Neumann) and positioned at an angle of 30°. The target area was identified using the surface vascularization as a guide. The pipette was monitored using a 4x objective (Olympus) and an eyepiece camera (DinoLite AM4023X). After the pipette was brought into the field of view (Figure 1B) we switched to a 16x or 40x objective (Nikon, Olympus, respectively) to image fluorescence (λ_excitation_ = 940 nm). A constant pressure of 250 mBar was maintained in the pipette. It was vertically displaced until reaching the dura, which caused a sudden increase in the pipette resistance to 30–40 MΩ. The pipette was then quickly moved back and forth along the axial axis of the manipulator until the pipette penetrated the dura and entered the brain, which was characterized by the resistance returning to baseline. The flow of Alexa was still clearly visible, ascertaining that the pipette was not clogged. The pipette was lowered along its diagonal axis to layer 2/3, i.e., between 150 and 400 μm, upon which pressure was decreased to 25 mBar. The time in between entering the brain and reducing the pressure was kept short (<30 s) in order to minimize damage to the tissue. The diffusion of Alexa made the cell bodies stand out as shadows. The pipette was then advanced toward a cell body until the resistance started to increase up to 30–50% of the baseline value, but not higher as this indicates that the pipette is pushing into the cell's membrane. The positive pressure was released to let the cell's membrane attach to the pipette, which further increased resistance. The DNA and Alexa were electroporated into the cell using a single pulse train (10 pulses, −12 V, 500 μs, 50 Hz). We avoided applying extra pulse trains, as this may cause damage. A successful electroporation resulted in a fast (in the order of 100 ms) filling of the cell body by Alexa (Figure 1C). On average this procedure was repeated three times per mouse (but no more than five). We used a single penetration tract and tried to minimize lateral movements of the pipette (<50 μm). Ideally all electroporations were done using a single pipette, but this was not always possible due to clogging. We were careful to keep the preparation clean and avoided biological and chemical contamination.

After removal of the pipette the dura was covered with sterile Gelfoam (Pfizer). The mouse was transferred back to the surgery setup. A sterile coverslip (#1, ∅ 3 mm) was implanted immediately as described previously (Holtmaat et al., 2009), with one difference: the coverslip was sunk into the craniotomy such that the surface of the glass was flush with the surface of the skull.

### *In Vivo* two photon laser scanning microscopy (2PLSM)

In between 1 to 5 days after the craniotomy we checked if cells expressed DsRed, without acquiring high-resolution images in order to avoid photodamage during the early stages of expression. Fifty-four out of 90 mice displayed fluorescence within this time frame: pCAG-DsRedExpress-WPRE and pCAG-PSD-95-eGFP-WPRE (five mice, 200 ng μl^−1^; two mice, 100 ng μl^−1^; 17 mice, 70 ng μl^−1^; three mice, 50 ng μl^−1^); pCAG-DsRedExpress-WPRE (four mice, 200 ng μl^−1^; two mice, 100 ng μl^−1^; two mice, 70 ng μl^−1^; two mice, 50 ng μl^−1^); pCAG-eGFP-gephyrin-WPRE and pCAG-DsRedExpress-WPRE (one mouse, 70 ng μl^−1^; seven mice, 50 ng μl^−1^); pCAG-eGFP-CaMKII (three mice, 50 ng μl^−1^); pCAG-SEP-GluR1-WPRE and pCAG-DsRedExpress-WPRE (four mice, 50 and 100 ng μl^−1^, respectively); hSyn1-mRuby2-GSG-P2A-GCaMP6s-WPRE-pA (two mice, 30 ng μl^−1^). After 10 days, the mice were inspected again and if the cells appeared healthy longitudinal imaging was started (except for the mRuby2 and GCaMP6s example, for which images were taken 2 days after SCE).

Imaging was performed using a custom-built 2PLSM (https://openwiki.janelia.org/wiki/display/shareddesigns/Shared+Two-photon+Microscope+Designs) and the data acquisition software package ScanImage (https://openwiki.janelia.org/wiki/display/ephus/ScanImage). For each imaging session, mice were anesthetized with MMF and placed under the microscope on a feedback controlled heating pad. As a light source for imaging, we used a tunable Ti:Sapphire laser (Chameleon Ultra II, Coherent) running at λ = 940 nm for simultaneous excitation of DsRedExpress and various green emitting fluorescent proteins. The power was typically between 80 and 120 mW at the back focal plane of the objective. The microscope was equipped with a 40x, 0.8 N.A. water immersion objective (LUMPFLN40XW, Olympus) and high quantum efficiency photomultiplier tubes (R3896, Hamamatsu). Green and red fluorescence were spectrally separated using a 565 nm dichroic mirror (565dcxr, Chroma) and two bandpass filters (HQ510/50m-2P and HQ620/60m-2P, Chroma). There was no DsRedExpress or GFP fluorescence bleed through across the two detection channels. Images were acquired at 2 ms/line (image size, 512 × 512 pixels for a typical field of view of 50 × 50 μm). Z-stacks were acquired with 1-μm steps and were composed of 50 to 200 frames. Imaging was repeated every day during the first week after the electroporation and every 8 days afterwards. For excitation of GCaMP6s and mRuby2, the laser was set at λ = 910 or 1040 nm, respectively, and image acquisition was performed using a 20x objective (0.95 NA, XLUMPLFL20XW/IR-SP, Olympus) and a GaAsP photomultiplier tube (10770PB-40, Hamamatsu). Focus shifts (< 260 nm) between the green and red signals due to chromatic aberration were negligible relative to the sizes of spines, dendrites and the spatial extent of the Ca^2+^ responses. Time-lapse images were acquired at 3.91 Hz (256 lines/frame, 1 ms/line). The average excitation power was kept below 40 mW, as measured at the focal plane of the objective.

### Image analysis

Gephyrin puncta were detected using custom-designed algorithm running in MATLAB (MathWorks, Inc.). Dendritic segments were traced in the red channel of 3D, mean-filtered (one-pixel radius), image stacks using the Simple Neurite Tracer (SNT) plugin (Longair et al., 2011) in FIJI (Schindelin et al., 2012). This trace was transferred to the green channel, and for each pixel along the trace the algorithm searched for the highest pixel value in the original image within an ellipse perpendicular to the axis of the trace, with a five-pixel radius along the major axis (image plane) and a two-pixel radius along the minor axis (across image planes). This typically spanned the width of dendritic shafts. If a pixel value was found to be higher than the original one of the trace, it was corrected. A rolling local baseline value was calculated from the mean of the 70% dimmest pixels found within a ±50-pixel window along the trace. For puncta detection we used a threshold of 2 standard deviations (2 SD) above the baseline. For volume corrections, a normalization factor was calculated by dividing the median pixel value of the trace in the red channel by the median pixel value of the trace in the green channel. Subsequently, each pixel of the trace in green trace was multiplied by this factor. This factor was also used to plot a normalized eGFP-Gephyrin image. Trace and image correction was achieved by pixel-by-pixel subtraction of the red from the normalized green values.

## Results

### Long-term expression and viability upon SCE

SCE was targeted to supragranular cells in the C2 barrel column, which was identified using intrinsic optical imaging (Figures 1A–C). On average, three cells were electroporated in each mouse (using a mixture of vectors encoding cytosolic and synaptic proteins). This resulted in one or two cells expressing fluorescence over the following days in 60% of the experiments. This percentage is negatively biased since these experiments included practicing rounds, various kinds of plasmids, and various DNA concentrations. Nonetheless, the electroporations never lead to visible disturbances of the dura and the underlying superficial vasculature (Figure 1D), similar to previous studies (Holtmaat et al., 2009). At first we used DNA concentrations that were previously shown to result in expression over 24 h (i.e., 70–200 ng μl^−1^) (Kitamura et al., 2008; Judkewitz et al., 2009). We found that some cells displayed pathological signs (blebby or fragmented dendrites) over the time course of 10 days. This may have also been due to issues related to the cranial window implantation. Therefore, the exact success rates of electroporation are difficult to assess. Not correcting for other confounding issues, we estimate that approximately 17% of the cells electroporated with 200 ng μl^−1^ were viable after 10 days. In general this percentage increased with lower DNA concentrations (25% for 100 ng μl^−1^; 42% for 70 ng μl^−1^; 46% for 50 ng μl^−1^). These results suggested that, although high DNA concentrations may be suitable for expression of cytosolic or physiologically inert proteins (Judkewitz et al., 2009), they are not well-tolerated by cells over long times when encoding synaptic proteins. Therefore, we settled at using a DNA concentration of 50 ng μl^−1^ (or lower). At this concentration cells could be imaged without obvious disturbances in the neurons' large-scale morphology (Figures 1E,F). We did not systematically test the efficiency of lower DNA concentrations. Expression was relatively stable over time (Figure 1E). Small differences in expression levels could have occurred, but these were difficult to assess due to variation in excitation and detection efficiencies at different time points, which depend on the optical properties of the cranial window prep (for discussion, see Holtmaat et al., 2009). This is of importance when analyzing synaptic protein aggregation and dynamics (e.g., see **Figure 4**).

### SCE-mediated expression of synaptic proteins

In order to illustrate the use of SCE for imaging synapses we expressed two postsynaptic scaffolding proteins (PSD-95 and gephyrin), as well as two postsynaptic plasticity markers for glutamatergic synapses (CaMKIIα and GluR1). PSD-95 is a PDZ-domain protein that binds to various postsynaptic components in most glutamatergic synapses, and modulates their function and maturation (Kim and Sheng, 2004). It is highly enriched in dendritic spines (Okabe et al., 1999). Gephyrin is present in most inhibitory synapses where it clusters glycine and GABA_A_receptors (Fritschy et al., 2008). CaMKIIα is a calmodulin dependent kinase that has been shown to act as a calcium oscillation decoder in neurons (De Koninck and Schulman, 1998). It plays a critical role in synaptic plasticity, and is activated and translocates to spines upon LTP (Okamoto et al., 2009; Lisman et al., 2012). GluR1 is a glutamate receptor subunit that is inserted into synapses upon strong synaptic stimulation (Shi et al., 1999; Kessels and Malinow, 2009; Huganir and Nicoll, 2013).

**Figure 2:**
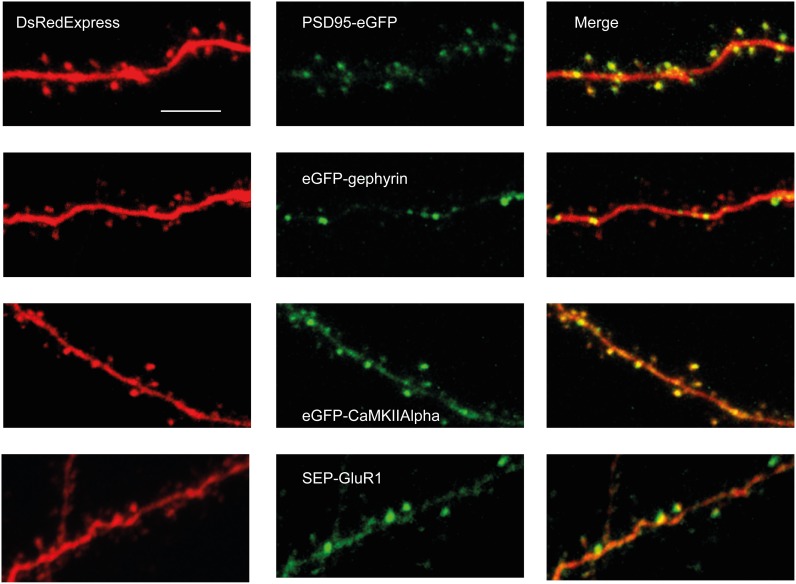
**Examples of GFP-tagged synaptic proteins co-expressed with DsRedExpress *in vivo***. 2PLSM-images (2PLSM-images (Maximum Intensity Projection of 5–20 single imaging planes) of PSD95-eGFP (first row), eGFP-Gephyrin (second row), eGFP-CaMKIIα (third row) and SEP-GluR1 (last row) through a chronic cranial window. All proteins display punctate distributions. Scale bar, 10 μm.

Upon SCE, DsRedExpress homogenously filled dendritic shafts and spines (Figure 2). In contrast, synaptic proteins did not homogeneously fill the cytosol, and were rather enriched in spines or formed clusters in the dendritic shaft (Figure 2). In neurons transfected with PSD-95-eGFP we observed clear puncta of various sizes in dendritic shafts and in dendritic spines, similar to experiments in organotypic slices (Okabe et al., 1999) or *in vivo* upon *in utero* electroporation (Cane et al., 2014) (Figure 2). We have previously shown that the puncta nearly perfectly overlap with asymmetric synapses, as detected using serial section electron microscopy (Cane et al., 2014). eGFP-gephyrin expression also resulted in a punctate labeling along dendrites. In accordance with studies using *in utero* electroporation (Chen et al., 2012; van Versendaal et al., 2012), the puncta were mostly found in dendritic shafts, and incidentally in spines (Figure 2). CaMKIIα was more diffusely distributed over dendrites (Figure 2). Nonetheless, there were clear hotspots in the dendritic shaft or in spines, similar to what has been found in organotypic slice cultures (Otmakhov et al., 2004). These clusters may represent synaptic locations that were recently activated and to which the protein has translocated (Lee et al., 2009). Similar to CaMKIIα, the expression of SEP-GluR1 resulted in a somewhat diffuse labeling with local accumulations of protein along dendrites and spines (Figure 2), which is similar to previous experiments in organotypic slices (Kopec et al., 2006; Patterson et al., 2010) and *in vivo* upon *in utero* electroporation (Makino and Malinow, 2011). These hotspots may represent synapses with a high rate of GluR1 subunit insertion (Ashby et al., 2004; Kopec et al., 2006; Patterson et al., 2010).

These examples demonstrate that SCE is able to drive expression of synaptic proteins, resulting in local fluorescent clusters that resemble the distribution of synapses. They are comparable to the results of studies using other gene transfer techniques in organotypic slices or *in vivo*.

### Imaging and analysis of synapse structural dynamics

**Figure 3:**
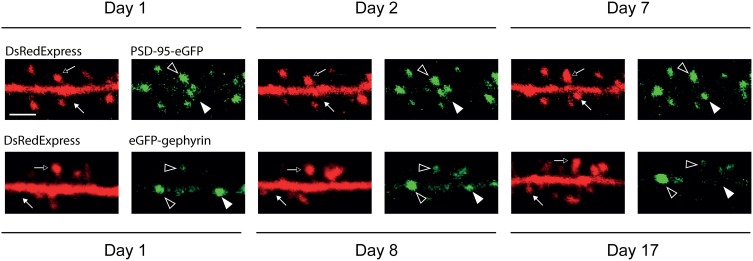
**Long-term expression and imaging of synaptic scaffold proteins**. Unfiltered time-lapse image examples of PSD-95-eGFP (first row) and eGFP-gephyrin (second row). Dendritic spines (arrows) are revealed by DsRedExpress. Putative excitatory (PSD-95) and inhibitory (gephyrin) postsynaptic elements (arrowheads) are in green. Some spines and synaptic puncta are persistent (open arrows and arrowheads) throughout the imaging period. Transient appearances (closed arrows and arrowheads) can be seen as well. Scale bar, 3 μm.

The synaptic scaffold proteins PSD-95 and gephyrin are reliable indicators of synapse size, and thereby form exquisite tools to study structural dynamics of synapses (Okabe et al., 1999, 2001; Friedman et al., 2000; Minerbi et al., 2009; Dobie and Craig, 2011; Woods et al., 2011; Chen et al., 2012; van Versendaal et al., 2012; Cane et al., 2014). SCE-transfected neurons expressing PSD-95-eGFP and eGFP-gephyrin could be imaged under a cranial window over days to weeks (Figure 3). Stable and labile fluorescent puncta could be observed.

We have previously shown that fluorescent puncta of auxiliary PSD-95-eGFP in L2/3 cells *in vivo* reflect the presence of glutamatergic synapses (Cane et al., 2014). Synaptic PSD-95-eGFP clusters can readily be distinguished from the dendritic PSD-95-eGFP pool since this protein strongly and preferentially binds to the synaptic scaffold (Kim and Sheng, 2004), which is large and contains on average more than 300 PSD-95 molecules (Sugiyama et al., 2005; Sheng and Hoogenraad, 2007). In addition, they mostly appear in spines. This spatially separates the bound molecules from the unbound molecules in the dendritic shaft. The fluorescence ratios between spines and shafts can be used as a measure of cluster size, and to estimate the fraction of diffusible molecules bound within spines (Otmakhov et al., 2004; Cane et al., 2014). As a benefit of these features, signals can easily be thresholded, which renders the puncta readily traceable over time (Okabe et al., 1999; Minerbi et al., 2009; Woods et al., 2011; Cane et al., 2014). Figure 3 shows an example of PSD-95-eGFP puncta at various time points in unfiltered images. These data confirm that some puncta are dynamic, whereas others are stable or persistent (Okabe et al., 1999, 2001; Friedman et al., 2000; Minerbi et al., 2009; Dobie and Craig, 2011; Woods et al., 2011; Cane et al., 2014).

**Figure 4:**
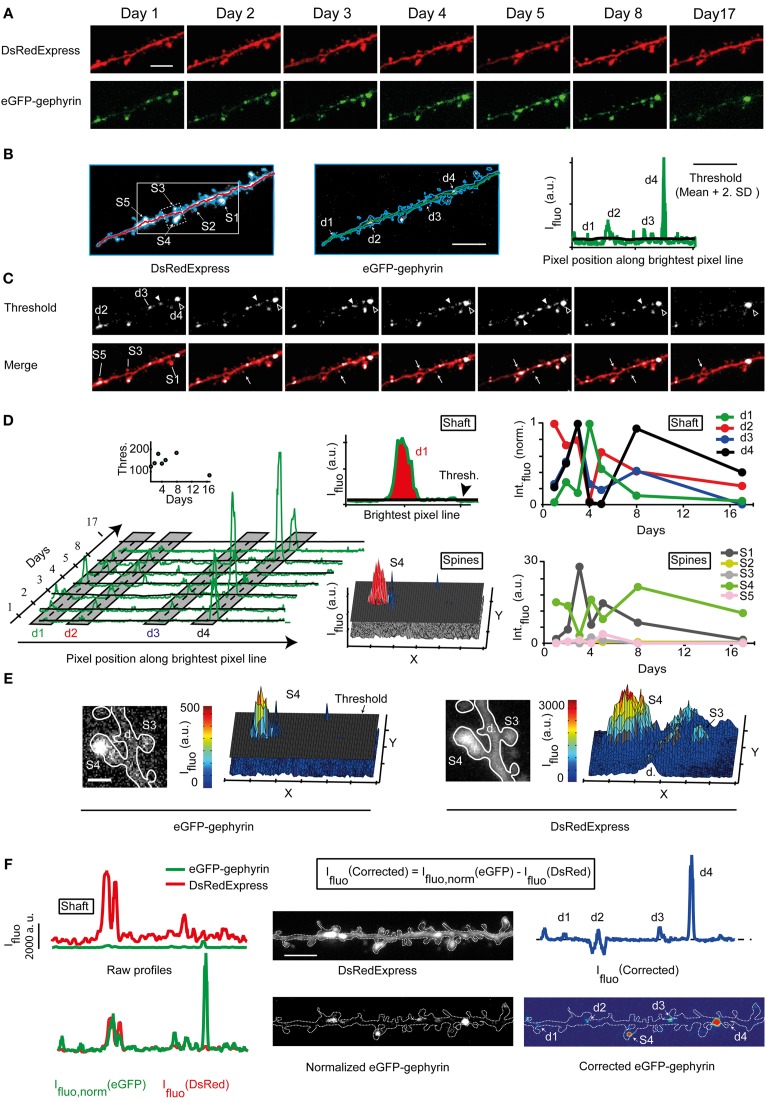
**Quantification of synaptic eGFP-Gephyrin puncta**. **(A)** Time lapse imaging of a portion of the dendrite expressing DsRedExpress and eGFP-Gephyrin. Scale bar, 6 μm. **(B)** Maximum pixel value projections of an image stack of a dendritic segment co-expressing DsRedExpress (left) and eGFP-Gephyrin (middle). Scale bar, 5 μm. The outline of this dendritic branch is in blue. The green and red lines connect the brightest pixels (in 3D) along the dendritic shaft in the image of the red channel. Right, the fluorescence intensity profile along this trace for the green channel. The threshold for puncta detection (black line) is defined as the baseline + 2xSD. Some examples of eGFP-gephyrin puncta with fluorescence intensities above threshold are marked (d1, d2, d3, and d4). **(C)** Time lapse images of the same dendrite as in **(A)**, but now thresholded in green to reveal the puncta. This image is blurred to remove single-pixel signals that are most likely not related to eGFP-gephyrin puncta. Dendritic puncta and spines are marked by “d” and “S,” respectively. Closed arrowheads, transient puncta. Open arrowheads, persistent puncta. White arrows, transient puncta in spines. **(D)** The temporal dynamics of eGFP-gephyrin puncta may be monitored from the intensity profiles on each time point (green lines and corresponding thresholds as black lines). Gray shadows illustrate the spatial window over which puncta are considered to be the same. The inset shows the mean threshold over time (left). The sizes of eGFP-gephyrin puncta are estimated by integrating the parts of the peaks above threshold (or the 2D integrated peaks above the threshold plane for spines) (middle). The examples illustrate that puncta dynamics can be tracked despite the fluctuations in ambient fluorescence levels (e.g., because of low threshold at day 17 puncta can still be detected). The fluorescence of several puncta fluctuates around threshold (right). **(E)** Images of a spine bearing an eGFP-gephyrin punctum and the corresponding 2D fluorescence profiles in the green (left) and red (right) channel. Scale bar, 1.5 μm. The threshold is indicated as a black sheet. **(F)** Raw (top left) and normalized (bottom left) spatial intensity profiles along the trace in the red and green channel. Based on this normalization the image of the green channel was normalized (middle) and the dendritic trace and image were corrected (right).

As compared to the PSD-95 in an excitatory synapse, the number of gephyrin molecules per synaptic cluster may be more than twice as low (Specht et al., 2013). They mostly appear along dendritic shafts (Dobie and Craig, 2011). Therefore, the auxiliary expression of eGFP-gephyrin may result in a relatively low contrast between the fluorescence that is present in puncta and the surrounding cytoplasm (Figure 4A). This complicates the thresholding of images. In addition, the lack of spatial segregation makes it harder to quantify cluster sizes and to track them over time.

To facilitate the unbiased scoring of eGFP-gephyrin puncta, we aimed at subtracting the ambient fluorescence levels from puncta fluorescence, assuming that the ambient (i.e., cytosolic) fluorescence reports the total expression levels of eGFP-gephyrin. We traced the shaft of the dendritic branch of interest in 3D on the DsRedExpress image stack (Figure 4B, left) using the Simple Neurite Tracer module in Fiji (Longair et al., 2011). This trace was transferred to the eGFP-gephyrin image stack (Figure 4B, middle), and used to plot an intensity profile of the brightest pixels in the dendritic shaft (Figure 4B, right; see Materials and Methods). Each eGFP-gephyrin fluorescence peak larger than a defined threshold (Figure 4B, right, black line; see Materials and Methods) was considered to represent a punctum. This procedure mainly detected puncta in the dendritic shaft or small spines protruding in the optical axis, since laterally protruding spines were usually not included in the trace. To track eGFP-gephyrin puncta over time each image was thresholded (Figure 4C). Peaks at subsequent time points that were located within a spatial window of 40 pixels relative to their initial position along the trace (Figure 4D, gray zones) were considered to be the same (e.g., the position of peaks d1, d2, d3, and d4 in Figure 4D slightly varied across time points). Peak integrals above threshold were plotted and used to estimate puncta brightness (Figure 4D, middle and right). This revealed that even though puncta could persist, their brightness varied over time. Some seemed to appear or disappear when their brightness exceeded or dropped below threshold (Figure 4D, right). Puncta in spines were detected separately using the mean value of the threshold (Figure 4D, middle and right, see Materials and Methods).

The above method allows a quick assessment of puncta brightness. However, puncta brightness may be overestimated in large volumes such as large spines or dilations in the dendrite. An example of this is given in Figure 4E. The spatial fluorescence profile of eGPF-gephyrin suggests that the spine contained a large “synaptic” cluster (Figure 4E, left). However, the profile of DsRedExpress also showed a peak in fluorescence, indicating that the volume of this spine was large (Figure 4E, right). To correct for variations in volume, the green intensity profile along the trace was normalized to the red signal (Figure 4F, left, see Materials and Methods). The green fluorescence profile remained distinct from the red fluorescence profile, indicating that the differences in eGFP-gephyrin fluorescence did not merely reflect variations in dendritic volume. To generate a corrected image of the green signal, the red image was subtracted from the normalized green image (Figure 4F, middle and right). The corrected profile in green indicates the “real” relationship between the peak eGFP-gephyrin intensities at various locations (Figure 4F, right).

### Imaging of proteins marking synaptic function

**Figure 5:**
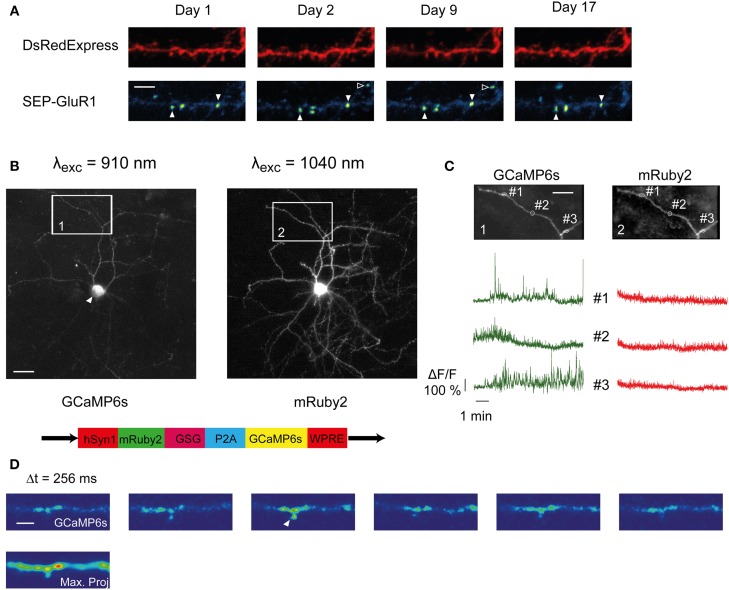
**SCE and longitudinal imaging of SEP-GluR1 and GCAMP6s**. **(A)** Unfiltered time-lapse images (maximum value projections) of a dendritic branch co-expressing SEP-GluR1 and DsRedExpress over 17 days. Some hotspots persistently appear at the same location (open arrowhead), others appear transiently (closed arrowhead). Scale bar, 5 μm **(B)** Maximum pixel value projection of an image stack of a neuron co-expressing GCaMP6s (left) and mRuby2 (right). The white arrowhead points toward the cell nucleus, which is not filled by GCaMP6s. Bottom, schematic of the bicistronic plasmid sequence (gift from Rose and Bonhoeffer). Scale bar, 15 μm **(C)** Gcamp6s (left) and mRuby (right) fluorescence dynamics in three ROIs from the boxed areas in **(B)**. **(D)** Time lapse image of GCaMP6s fluorescence along a dendritic segment. The time interval between two successive frames is 256 ms. The Ca^2+^ transient in the spine likely represents synaptic input (arrowhead). Scale bar, 3 μm.

Enrichment of GFP-tagged GluR1 in the postsynaptic membrane can be seen upon the induction of LTP (Shi et al., 1999), and is thought to reflect synaptic strengthening (Kessels and Malinow, 2009; Huganir and Nicoll, 2013). Using GFP-tagged receptors, GluR1-enriched synapses are hard to distinguish, since the fraction of GluR1 stored in vesicles is relatively high under baseline conditions. Recently, super ecliptic pHluorin-tagged GluR1 (SEP-GluR1) has been used to facilitate the visualization of GluR1 dynamics (Ashby et al., 2004; Kopec et al., 2006; Patterson et al., 2010; Makino and Malinow, 2011). In these constructs the ecliptic pHluorin (a pH-sensitive form of GFP) is tagged to the N-terminus of the receptor. Upon activity-mediated exocytosis the fluorophore translocates from the acidic environment of the vesicles to a neutral pH in the extracellular space, resulting in a strong increase of fluorescence (Miesenböck et al., 1998). Therefore, the SEP-GluR1 fluorescence provides a direct measure of the rate of GluR1 receptor subunit exocytosis (Ashby et al., 2004; Kopec et al., 2006; Patterson et al., 2010). We longitudinally imaged SEP-GluR1 after SCE (Figure 5A). On each time point we detected various hotspots, presumably representing synapses that were activated just before imaging. Interestingly, some hotspots were repeatedly seen at the same location, which suggests that some synapses may be persistently activated and undergo constitutive GluR1 insertion under baseline conditions.

Genetically encoded calcium indicators (GECIs) are used to longitudinally track synaptic activity *in vivo* (Tian et al., 2009; Chen et al., 2013). We tested if SCE can be used to express GECIs. We electroporated a bicistronic expression vector encoding mRuby2 and GCaMP6s, linked by a P2A cleavage peptide (Addgene plasmid # 50942) (Figure 5B, bottom). This resulted in readily detectable mRuby2 fluorescence (Figure 5B, right). GCaMP6s was usually much dimmer (Figure 5B, left), which may be due to the low resting fluorescence of GCaMP6 (Chen et al., 2013). The spatial distribution of GCaMP6s within the cell was similar to what has been reported (Chen et al., 2013). The cell nuclei remained largely spared (Figure 5B, white arrowhead), whereas in the rest of the cell the distribution resembled that of mRuby2 (Figure 5B, compare left and right).

We observed localized and spontaneous fluctuations in GCaMP6s fluorescence in the dendritic tuft. Neurons located in supragranular layers of the barrel cortex are known to display spontaneous local Ca^2+^ transients (Svoboda et al., 1997; Gambino et al., 2014; Palmer et al., 2014). Imaging of small dendritic regions (~1–2 μm) with high temporal resolution (3.91 Hz) under wakefulness revealed similar transients (Figure 5C left). Simultaneously recorded mRuby2 fluorescence amplitudes were considerably smaller (mRuby2, δF/F mean = 0.099 ± 0.17, max = 0.94; GCaMP6s, δF/F mean = 0.215 ± 0.36, max = 3.83), confirming that the dynamics were not due to movement artifacts, and likely reflected Ca^2+^ transients. They could also be observed in and around dendritic spines (Figure 5D, arrowhead), presumably reflecting excitatory synaptic activity. This indicates that GCaMP6s can be used to image Ca^2+^ in small dendritic compartments in combination with cytosolic mRuby2.

Taken together these results indicate that SCE provides a useful tool to express and track markers of synaptic activity and plasticity in layer 2/3 neurons *in vivo*.

## Discussion

Here we have provided a proof of principle for combining SCE of L2/3 neurons in the mouse neocortex with long-term 2-PLSM of synaptic proteins *in vivo*. In a substantial portion of the cells that were successfully transfected, synaptic structures could be imaged over several weeks. Over this time frame the surrounding tissue and cell morphology were not visibly affected. Spines did display turnover, in accordance with previous work (Trachtenberg et al., 2002; Holtmaat et al., 2005; Chen et al., 2012; van Versendaal et al., 2012; Cane et al., 2014). We did not quantify and systematically compare turnover rates. Previously we have studied PSD-95-eGFP dynamics and showed that SCE-mediate expression of PSD-95-eGFP generated fluorescent puncta that exactly matched the presence of asymmetric synapses as detected using electron microscopy (Cane et al., 2014). Here we show, in addition, that the auxiliary expression of eGFP-Gephyrin, eGFP-CaMKIIα, and SEP-GluR1 using SCE results in punctate labeling in dendritic shafts and spines, analogous to the distribution of postsynaptic elements. We observed stable and dynamic eGFP-gephyrin puncta, similar to the PSD-95 experiments (Cane et al., 2014) and other gephyrin studies (Chen et al., 2012; van Versendaal et al., 2012) *in vivo*. In our experience, the manual scoring of eGFP-gephyrin-puncta dynamics was less straightforward than scoring of PSD-95-eGFP puncta, since they were of lower contrast and mainly located in dendritic shafts. Therefore, tracking of eGFP-gephyrin puncta over time is best done based on spatial intensity profiles. We showed for a small number of puncta that such an unbiased analysis is useful for revealing eGFP-gephyrin dynamics. The method can be further improved by normalization of eGFP-gephyrin-derived pixel values to red fluorescence, which corrects for variation in dendritic or spine volumes. Retrospective electron microscopy will be needed to verify which threshold renders puncta representing GABAergic synapses with high fidelity, and detects the appearance and disappearance of synapses.

We showed that SCE could also be used to express and longitudinally image SEP-GluR1 dynamics. We observed hotspots that were likely representing highly active synapses displaying high exocytosis levels of vesicles containing GluR1 subunits (Ashby et al., 2004; Kopec et al., 2006; Patterson et al., 2010). Interestingly, some hot spots appeared at identical positions (spines) along the dendrite, suggesting that some synapses are highly active under baseline conditions (at least at the time of imaging) (Makino and Malinow, 2011). Other spots transiently appeared which may represent incidental synaptic strengthening. Imaging of GCaMP6s also revealed hotspots, presumably generated by spontaneous, synaptically evoked Ca^2+^ transients. It will be interesting to see whether some Ca^2+^ transients also persistently appear at identical locations over time. This would confirm the GluR1 experiments, which suggested that some synapses display high levels of spontaneous activity and therefore constitutively insert GluR1 receptors.

### Technical considerations

In our experience, the SCE method, combined with long-term imaging, bears various technical issues that need some consideration. The electroporation itself can be harmful for neurons, as discussed previously (Kitamura et al., 2008; Judkewitz et al., 2009). As a result, not all electroporated cells will survive until the first imaging time point. To increase success rates several cells can be electroporated (Judkewitz et al., 2009). However, it should be noted that increasing the cell numbers will take more time, which may reduce the probability to obtain a clear cranial window.

We experienced that the electroporation of DNA at high concentrations (e.g., 200 ng.μl^−1^) damaged neurons over subsequent days. These concentrations have been shown to suit well the visualization of GFP within the first day after electroporation (Judkewitz et al., 2009). However, in our case strong overexpression of the synaptic proteins may have resulted in dominant negative interference with endogenous synaptic proteins, or produced artificial and harmful protein aggregates. This raises the question as to what are the lowest DNA concentrations that minimally impact synaptic function; yet produce sufficient levels of fluorescence for imaging *in vivo*. We went as low as 30–50 ng.μl^−1^, which provided a reasonable throughput and did not produce obvious changes in dendritic morphology. Nonetheless, this concentration may not yet be optimal, and may have impacted the neurons' physiology. Indeed, cytosolic protein levels of PSD-95-eGFP and eGFP-gephyrin may have been higher than reported upon *in utero* electroporation (Chen et al., 2012; van Versendaal et al., 2012). Since we did not further characterize the physiological properties of the transfected neurons, we cannot be certain that 50 ng.μl^−1^ DNA is a “safe” concentration. Nonetheless, the possible impact of protein overexpression on the neuronal physiology does not distinguish the technique from most other transfection techniques. In fact, in contrast to many other techniques, SCE allows one to search for conditions leading to optimal expression levels with higher turnaround times than most other transfection techniques. However, the best way to avoid any interference with synaptic function would be to generate a locus-specific knock in that renders the endogenous pool of synaptic proteins fluorescent in a conditional manner (Fortin et al., 2014).

Despite the relatively low throughput, SCE has some distinct advantages (see Kitamura et al., 2008; Judkewitz et al., 2009 for extensive discussion). Similarities and differences with other transfection techniques in the neocortex are given in Supplementary Table 1. The most important advantage of SCE is the ability to target expression to a specific location or cell type. As shown here, it allowed for imaging of a single neuron in a predetermined cortical column. This may facilitate the comparison of data across mice, since all cells would be located in the same cortical environment. In addition, when combined with cell-specific fluorescent transgenic mouse lines, particular cell types could be targeted under visual (2-PLSM) guidance, and studied in a highly reproducible manner, even in infragranular layers (Andrásfalvy et al., 2014). Temporal control of expression could be improved by the use of conditional promoters, similar to approaches taken to optimize the *in utero* electroporation method (Ako et al., 2011). SCE potentially provides means to force co-express proteins of arbitrary sizes. For example, here we electroporated pCAG-SEP-GLUR1-WPRE, which size (5.7 kb) exceeds the packaging limit of AAV (4.7 kb) (Wu et al., 2010). Theoretically, SCE also allows transfection of RNA vectors or oligonucleotides that would otherwise demand complex vector systems. The labeling of a single neuron in a completely naïve background is another advantage. It ascertains that any labeled structure (e.g., a remotely located axonal element) is derived from the neuron of interest. In addition, due to the high fluorescence contrast it allows imaging of subcellular structures with high spatial resolution. Indeed, in our experiments the low background fluorescence facilitated imaging of SEP-GluR1, a protein that usually yields very low fluorescence.

### Conflict of interest statement

The authors declare that the research was conducted in the absence of any commercial or financial relationships that could be construed as a potential conflict of interest.
