# Corrigendum: Single cell electroporation for longitudinal imaging of synaptic structure and function in the adult mouse neocortex *in vivo*

**DOI:** 10.3389/fnana.2015.00056

**Published:** 2015-05-12

**Authors:** Stéphane Pagès, Michele Cane, Jérôme Randall, Luca Capello, Anthony Holtmaat

**Affiliations:** ^1^Department of Basic Neurosciences and the Center for Neuroscience, Centre Médical UniversitaireGeneva, Switzerland; ^2^Itopie Informatique, Société CoopérativeGeneva, Switzerland

**Keywords:** Single cell electroporation, *in vivo*, Long-term imaging, calcium imaging, dendritic spine

## Conflict of interest statement

The authors declare that the research was conducted in the absence of any commercial or financial relationships that could be construed as a potential conflict of interest.

