# Supplementary material for: Single cell electroporation for longitudinal imaging of synaptic structure and function in the adult mouse neocortex in vivo
Source: Front Neuroanat. 2015 Apr 7;9:36. doi: 10.3389/fnana.2015.00036 (PMC4387926; doi:10.3389/fnana.2015.00036)
Supplement: Supplementary file 1 [file Table1.DOCX]

|  | **Single Cell Electroporation** | **In Utero Electroporation** | **Viral Vectors** |
| --- | --- | --- | --- |
| **Control over temporal expression pattern** | - Onset of expression can be as fast as 1 day after transfection - Expression can be delayed or made conditional by various transcriptional promoters | - Onset of expression can be as fast as 1 day after transfection - Expression can be delayed or made conditional by various transcriptional promoters | - Onset of expression determined by the virus type (within days to weeks) - Expression can be delayed or made conditional by various transcriptional promoters |
| **Control over spatial expression pattern** | - Anatomical location can be precisely selected - Expression can be limited to a single neuron - Expression maximally in 10s of cells, in various layers | - Anatomical location can roughly be selected (spread over several 100s of μm) - Expression can be limited to a few cells, but usually several hundreds - Expression can be massive, limited to one layer | - Anatomical location can be roughly selected (spread over >100 μm) - Expression can be limited to several hundreds of cells - Expression can be massive, spreading over various layers |
| **Duration of experimental procedures** | - Ordering of adult mice (~days) - ~2 h for electroporation and cranial window - Occasional check for expression - ~10 d before imaging | - Generation or ordering of timed pregnancies (~weeks) - ~1 h for electroporation - Check of expression in newborns - ~4 w to have adult mice - ~1 h for cranial window - ~10 days before imaging | - Ordering of adult mice (~days) - ~ 1.5h for stereotaxic injection and cranial window - Waiting time for expression (days to weeks, depending on vector type) and imaging |
| **Skills and equipment required** | - Experience with in vivo electrode manipulation - Craniotomy and cranial window experience - Electroporator and headstage; electrode puller; micro manipulators; microscope | - Surgical experience; handling embryos in utero; - Craniotomy and cranial window experience - Electroporator; injection equipment; electrode puller; surgery macroscope | - Experience with stereotaxic injections - Craniotomy and cranial window experience - Stereotact; nanoinjector; electrode puller; surgery macroscope |
